# Over-activation of AKT signaling leading to 5-Fluorouracil resistance in SNU-C5/5-FU cells

**DOI:** 10.18632/oncotarget.24952

**Published:** 2018-04-13

**Authors:** Eun-Ji Kim, Gyeoung-Jin Kang, Jung-Il Kang, Hye-Jin Boo, Jin Won Hyun, Young Sang Koh, Weon-Young Chang, Young Ree Kim, Jung-Mi Kwon, Young Hee Maeng, Eun-Sook Yoo, Chang Hoon Lee, Hee-Kyoung Kang

**Affiliations:** ^1^ College of Pharmacy, Dongguk University, Seoul 04620, Republic of Korea; ^2^ Department of Medicine, School of Medicine, Jeju National University, Jeju 63243, Republic of Korea; ^3^ Creative Research Initiative Center for Concurrent Control of Emphysema and Lung Cancer, College of Pharmacy, Seoul National University, Seoul 08826, Republic of Korea; ^4^ Department of Internal Medicine, Jeju National University Hospital, Jeju 63241, Republic of Korea

**Keywords:** SNU-C5/5-FU, 5-Fluorouracil resistance, over-activation of AKT, E-cadherin, COX-2

## Abstract

Here, we investigated whether over-activation of AKT pathway is important in the resistance to 5-fluorouracil (5-FU) in SNU-C5/5-FU cells, 5-FU-resistant human colon cancer cells. When compared to wild type SNU-C5 cells (WT), SNU-C5/5-FU cells showed over-activation of PI3K/AKT pathway, like increased phosphorylation of AKT, mTOR, and GSK-3β, nuclear localization of β-catenin, and decreased E-cadherin. Moreover, E-cadherin level was down-regulated in recurrent colon cancer tissues compared to primary colon cancer tissues. Gene silencing of AKT1 or treatment of LY294002 (PI3 kinase inhibitor) increased E-cadherin, whereas decreased phospho-GSK-3β. LY294002 also reduced protein level of β-catenin with no influence on mRNA level. PTEN level was higher in SNU-C5/WT than SNU-C5/5-FU cells, whereas the loss of PETN in SNU-C5/WT cells induced characteristics of SNU-C5/5-FU cells. In SNU-C5/5-FU cells, NF-κB signaling was activated, along with the overexpression of COX-2 and stabilization of survivin. However, increased COX-2 contributed to the stabilization of survivin, which directly interacts with cytoplasmic procaspase-3, while the inhibition of AKT reduced this cascade. We finally confirmed that combination treatment with 5-FU and LY294002 or Vioxx could induce apoptosis in SNU-C5/5-FU cells. These data suggest that inhibition of AKT activation may overcome 5-FU-resistance in SNU-C5/5-FU cells. These findings provide evidence that over-activation of AKT is crucial for the acquisition of resistance to anticancer drugs and AKT pathway could be a therapeutic target for cancer treatment.

## INTRODUCTION

Colon cancer is known to be a significant cause of cancer morbidity and mortality. As the incidence of colon cancer has increased, a variety of treatments have been developed to treat it. Nevertheless, acquired resistance to anticancer drugs leads to therapy failure, and in fact 80% of cancer-related deaths are related to resistance to anticancer drugs [1, 2]. To surmount anticancer drug resistance, and achieve effective therapy, it is important to examine closely the mechanisms by which cells become resistant to anticancer drugs.

5-Fluorouracil (5-FU), which prevents DNA synthesis by targeting thymidylate synthase (TS), is commonly used as an anti-cancer drug to treat colorectal cancer [3]. An increase in the expression of TS is well-known to be a resistance mechanism to 5-FU treatment. However, a recent study has reported that SNU-C5/5-FU, fluorouracil-resistant human colon cancer cells which have acquired resistance to 5-FU, did not have increased expression of TS, suggesting that another mechanism of resistance to 5-FU exists [4].

The phosphatidylinositol 3-kinase (PI3K)/AKT pathway regulates a variety of cellular processes, including cell growth and survival [5]. In addition, it has been reported that activated AKT promotes Wnt/β-catenin signaling [6], and induces the instability of E-cadherin by activating mdm2, an anti-apoptotic protein [7]. In addition, AKT activation modulates NF-κB signaling through the activation of IκB kinase (IKK), thereby promoting survival and resistance to apoptosis in cancer cells [8–10]. AKT activation is strongly related to the inactivation of the tumor suppressor gene phosphatase and tensin homolog (PTEN) as result of its deletion on chromosome ten [11]. Therefore, activation of AKT contributes to maintaining the immortality of cancer cells by regulating the activity of various apoptosis-related proteins, even when cancer cells are exposed to stressful conditions, such as occur during anticancer drug treatment [11, 12].

Cyclooxygenase-2 (COX-2) is an inducible enzyme that is found to be upregulated in inflammation and cancer [13, 14]. Increased COX-2 levels are associated with cell survival and tumor development [15, 16], as well as resistance to apoptosis [17, 18]. In SNU-C5/5-FU cells, the levels of COX-2 and PGE_2_ have been found to be increased. Recent studies have reported that survivin, a member of the inhibitor of apoptosis (IAP) family, is stabilized by PGE_2_, which suppresses its ubiquitination [19]. Over-expression of survivin has been suggested to contribute to resistance to anti-cancer drugs, as well as to radiation therapy [20].

Here, we suggest a therapeutic strategy to overcome anticancer drug resistance, by examining the mechanism of 5-FU resistance in SNU-C5/5-FU cells.

## RESULTS

### Differences in the activation of the AKT and β-catenin pathways between SNU-C5/WT and SNU-C5/5-FU cells

**Figure 1:**
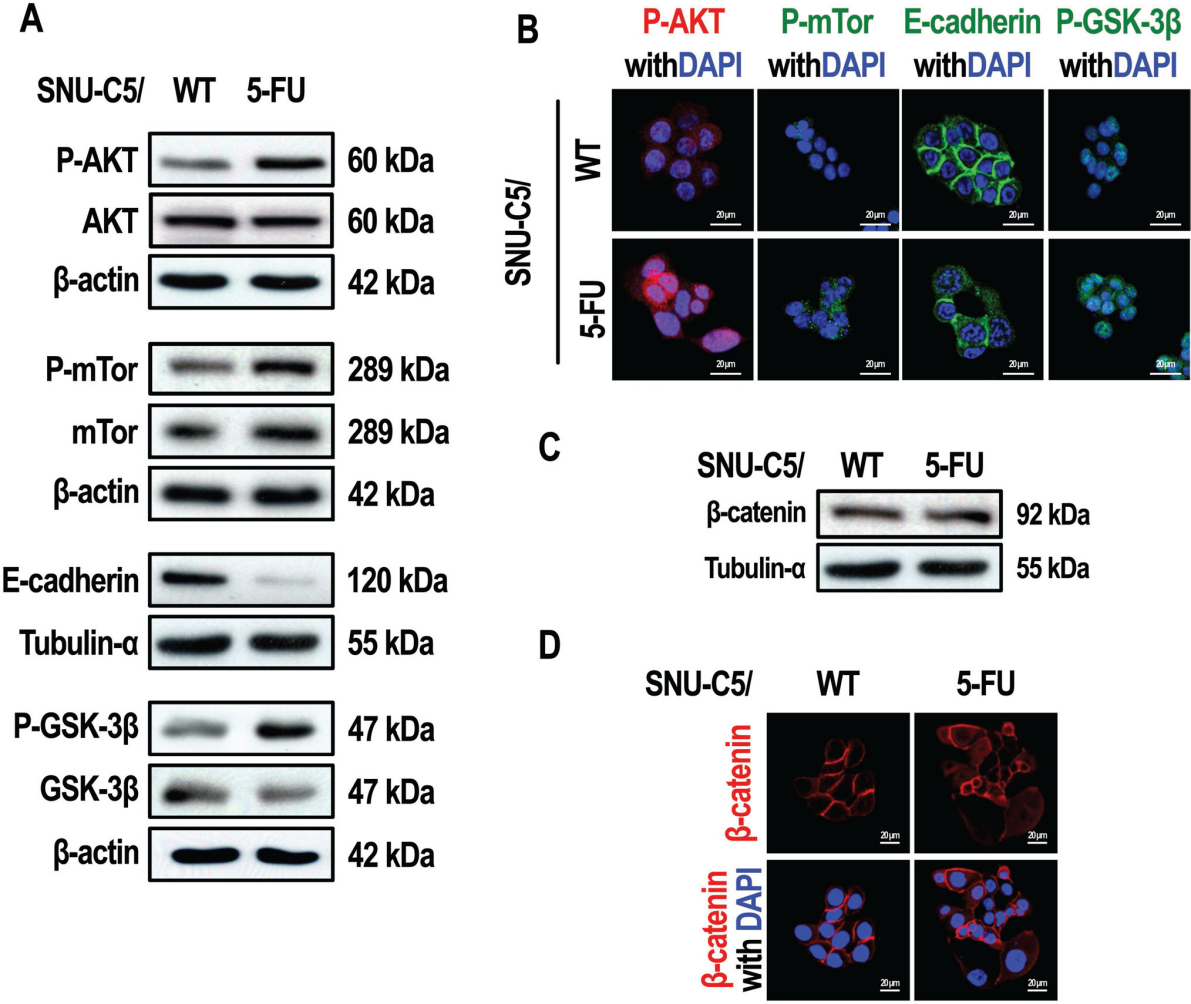
SNU-C5/5-FU cells have a markedly different phenotype compared to the SNU-C5/WT parental cells (**A**) Immunoblot analysis of P-AKT, AKT, P-mTOR, mTOR, E-cadherin, P-GSK-3β, and GSK-3β in SNU-C5/WT and SNU-C5/5-FU cells. (**B**) The localization of P-AKT, P-mTOR, E-cadherin, and P-GSK-3β was confirmed by confocal microscopy. Scale bars, 20 μm. (**C**) Immunoblot analysis of β-catenin levels in SNU-C5/WT and SNU-C5/5-FU cells. (**D**) The nuclear localization of β-catenin was confirmed by confocal microscopy. Scale bars, 20 μm.

Activation of the PI3K/AKT pathway can lead to resistance to apoptosis in cancer cells [21]. To examine whether the AKT pathway is activated in anticancer drug-resistant cells, we compared the levels of phospho-AKT in SNU-C5/5-FU cells with the same levels in the wild type parental cell line SNU-C5/WT. Interestingly, phospho-AKT levels were higher in SNU-C5/5-FU cells than SNU-C5/WT cells (Figure 1A and 1B). mTOR, a protein modulated by phospho-AKT [22], was also found to have increased phosphorylation in SNU-C5/5-FU cells compared with SNU-C5/WT cells (Figure 1A and 1B). Various growth factors are known to activate the PI3K/AKT pathway, resulting in the phosphorylation of GSK-3β and an increase in the transcriptional activity of β-catenin [23, 24]. β-catenin is also involved in cell-cell adhesion because of its interaction with E-cadherin in the plasma membrane [25, 26]. In contrast, activation of AKT leads to a decrease in E-cadherin expression [7]. This loss of E-cadherin expression leads to an increase in cytoplasmic β-catenin [27] and its rapid elimination through a process mediated by GSK-3β. If GSK-3β is phosphorylated and inactivated, β-catenin is instead translocated to the nucleus where it acts as a transcription factor [28]. An examination of the levels of both E-cadherin and phospho-GSK-3β, revealed that the levels of E-cadherin were lower, whereas the levels of phospho-GSK-3β were higher, in SNU-C5/5-FU cells compared to SNU-C5/WT cells (Figure 1A and 1B). When the expression and location of β-catenin were examined, we found no significant difference in the expression of β-catenin in whole cell lysates of SNU-C5/5-FU cells compared with lysates of SNU-C5/WT cells (Figure 1C). However, nuclear β-catenin levels were higher in SNU-C5/5-FU cells than in SNU-C5/WT cells (Figure 1D).

**Figure 2:**
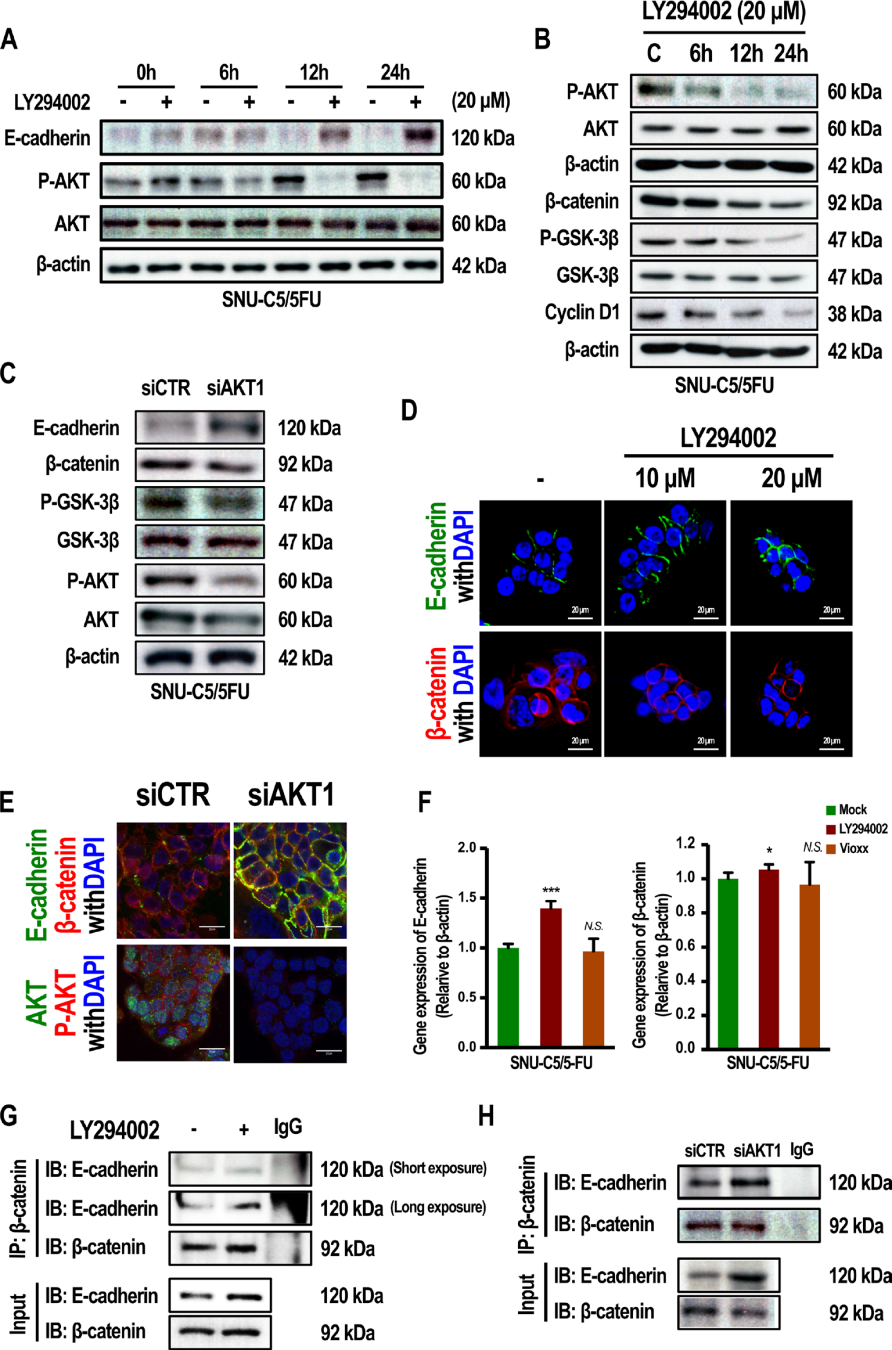
Inhibition of PI3K/AKT or gene silencing of AKT1 modulate the phenotype of SNU-C5/5-FU cells (**A**) Immunoblot analysis of E-cadherin, P-AKT, and AKT in SNU-C5/5-FU cells treated with LY294002 (20 μM). (**B**) Immunoblot analysis of P-AKT, AKT, β-catenin, P-GSK-3β, GSK-3β, and cyclin D1 in SNU-C5/5-FU cells treated with LY294002 (20 μM). (**C**) For gene silencing of AKT1, SNU-C5/5-FU cells were transfected with the indicated amount of AKT1 siRNA or control siRNA. Immunoblot analysis of E-cadherin, P-GSK-3β, GSK-3β, P-AKT, and AKT in SNU-C5/5-FU cells (**D**) The localization of E-cadherin and β-catenin were confirmed by confocal microscopy. Scale bars, 20 μm. (**E**) For gene silencing of AKT1, SNU-C5/5-FU cells were transfected with the indicated amount of AKT1 siRNA or control siRNA. The localization of E-cadherin, β-catenin, AKT and P-AKT were confirmed by confocal microscopy. Scale bars, 20 μm. (**F**) Real-time PCR (qPCR) measured E-cadherin and β-catenin mRNA levels in SNU-C5/5-FU cells treated with the LY294002 (20 μM) or Vioxx (20 μM). (**G** and **H**) Co-immunoprecipitation (IP) of β-catenin with E-cadherin in SNU-C5/5-FU cells.

To address the question of whether levels of E-cadherin, and phospo-GSK-3β depend on AKT activation, SNU-C5/5-FU cells were treated with LY294002, a well-known PI3 kinase inhibitor. As a result, E-cadherin expression increased by LY294002 treated in a time-dependent manner, whereas E-cadherin levels were reduced or remained low in the condition of no-LY294002. (Figure 2A). The phosphorylation of mTOR was reduced by LY294002 in a time-dependent manner (Supplementary Figure 1A). Treatment with LY294002 also resulted in a decrease in the levels of phospho-GSK-3β, which was followed by down-regulation of β-catenin and cyclin D1 (Figure 2B and Supplementary Figure 1B). Gene silencing of AKT1 also suppressed phospho-GSK-3β and induced level of E-cadherin (Figure 2C). These data were supported by a confocal study. Figure 2D shows that treatment with LY294002 decreased the level of β-catenin in the nucleus, whereas the levels of E-cadherin increased. β-catenin interacts with E-cadherin at the plasma membrane and is involved in cell-cell adhesion [25, 26]. Gene silencing of AKT1 increased the binding of E-cadherin and β-catenin (Figure 2E and 2H). Moreover, when SNU-C5/5-FU cells were treated with LY294002, the interaction of β-catenin with E-cadherin increased (Figure 2G). Similar to the Western blot results, treatment of LY294002 increased mRNA level of E-cadherin in SNU-C5/5-FU cells (Figure 2F) and SNU-C5/WT (Supplementary Figure 1C). While protein level of β-catenin was reduced by LY294002 (Figure 2B), mRNA level of β-catenin was not reduced by LY294002 in SNU-C5/5-FU (Figure 2F). Interestingly, mRNA level of β-catenin was reduced by LY294002 in SNU-C5/WT cells (Supplementary Figure 1C). These results show that LY294002 regulates β-catenin mainly at the protein level in SNU-C5/5-FU cells. These data suggest that activation of AKT regulates the β-catenin pathway along with E-cadherin expression in SNU-C5/5-FU cells.

### Silencing of PTEN results in the activation of AKT in SNU-C5/WT cells

**Figure 3:**
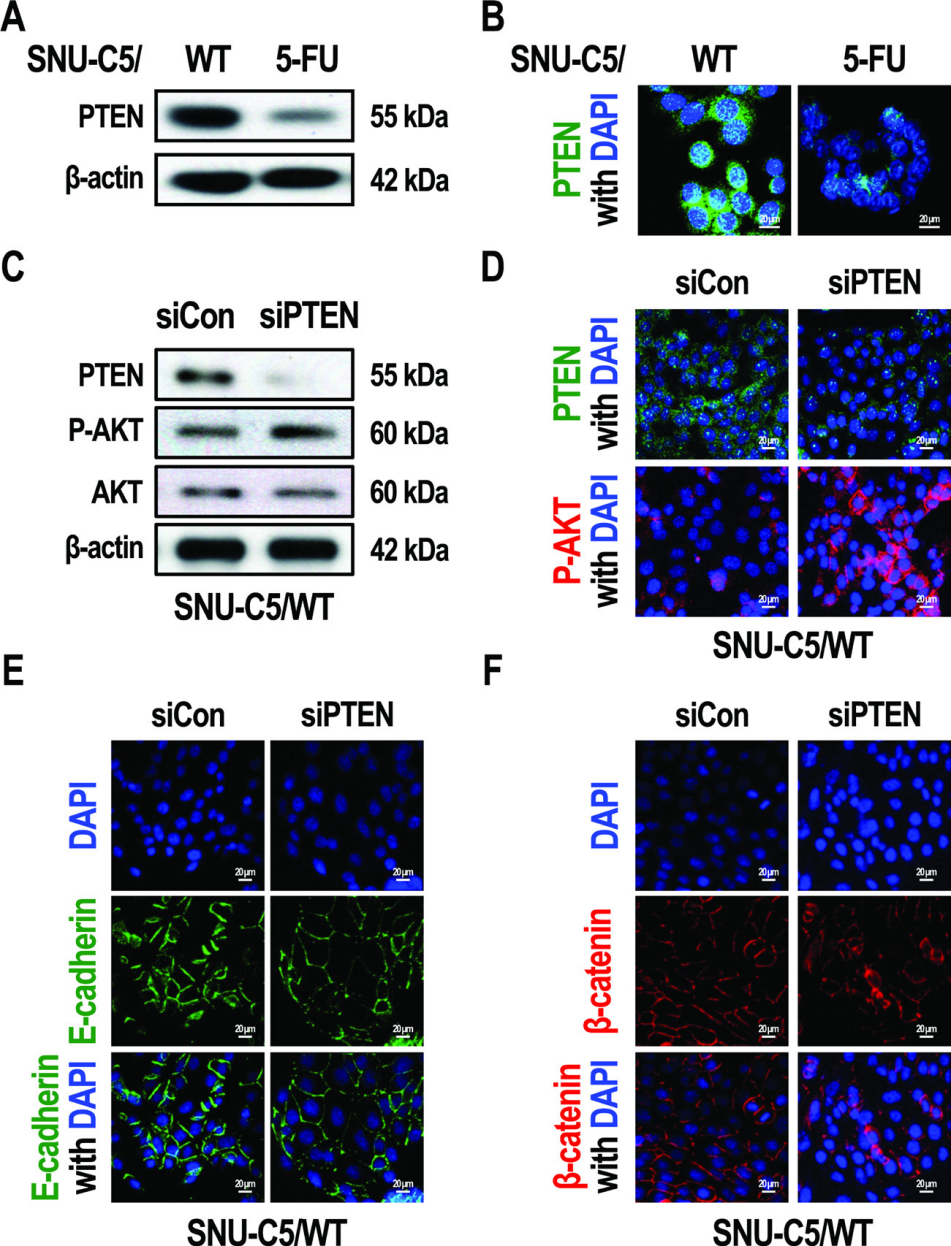
Silencing of PTEN allows SNU-C5/WT cells to adopt the phenotype of SNU-C5/5-FU cells (**A**) Immunoblot analysis of PTEN in SNU-C5/WT and SNU-C5/5-FU cells. (**B**) The localization of PTEN was confirmed by confocal microscopy. Scale bars, 20 μm. (**C**) Immunoblot analysis of PTEN, p-AKT, and AKT in si-PTEN transfected SNU-C5/WT cells. (**D**) The localization of PTEN and p-AKT was confirmed by confocal microscopy. Scale bars, 20 μm. (**E**) The localization of E-cadherin was confirmed by confocal microscopy. Scale bars, 20 μm. (**F**) The localization of β-catenin was confirmed by confocal microscopy. Scale bars, 20 μm.

The loss of PTEN has been reported to promote the phosphorylation of AKT in colon cancer [29]. We examined PTEN levels and found that PTEN expression was decreased in SNU-C5/5-FU cells compared to SNU-C5/WT cells (Figure 3A and 3B). To address whether this down-regulation of PTEN could induce the phenotype seen in SNU-C5/5-FU cells, we silenced PTEN expression in SNU-C5/WT cells. Treatment of SNU-C5/WT cells with an siRNA targeted to PTEN resulted in an increase in AKT phosphorylation (Figure 3C and 3D), which was followed by a decrease in E-cadherin expression (Figure 3E). Silencing of PTEN in SNU-C5/WT cells also led to an increase in β-catenin expression in the nucleus compared to the plasma membrane (Figure 3F). These data suggest that silencing of PTEN in SNU-C5/WT cells seems to mimic the phenotype seen in SNU-C5/5-FU cells, such as increased AKT phosphorylation, decreased E-cadherin expression, and increased levels of nuclear β-catenin.

### Activation of AKT leads to the activation of NF-κB in SNU-C5/5-FU cells

**Figure 4:**
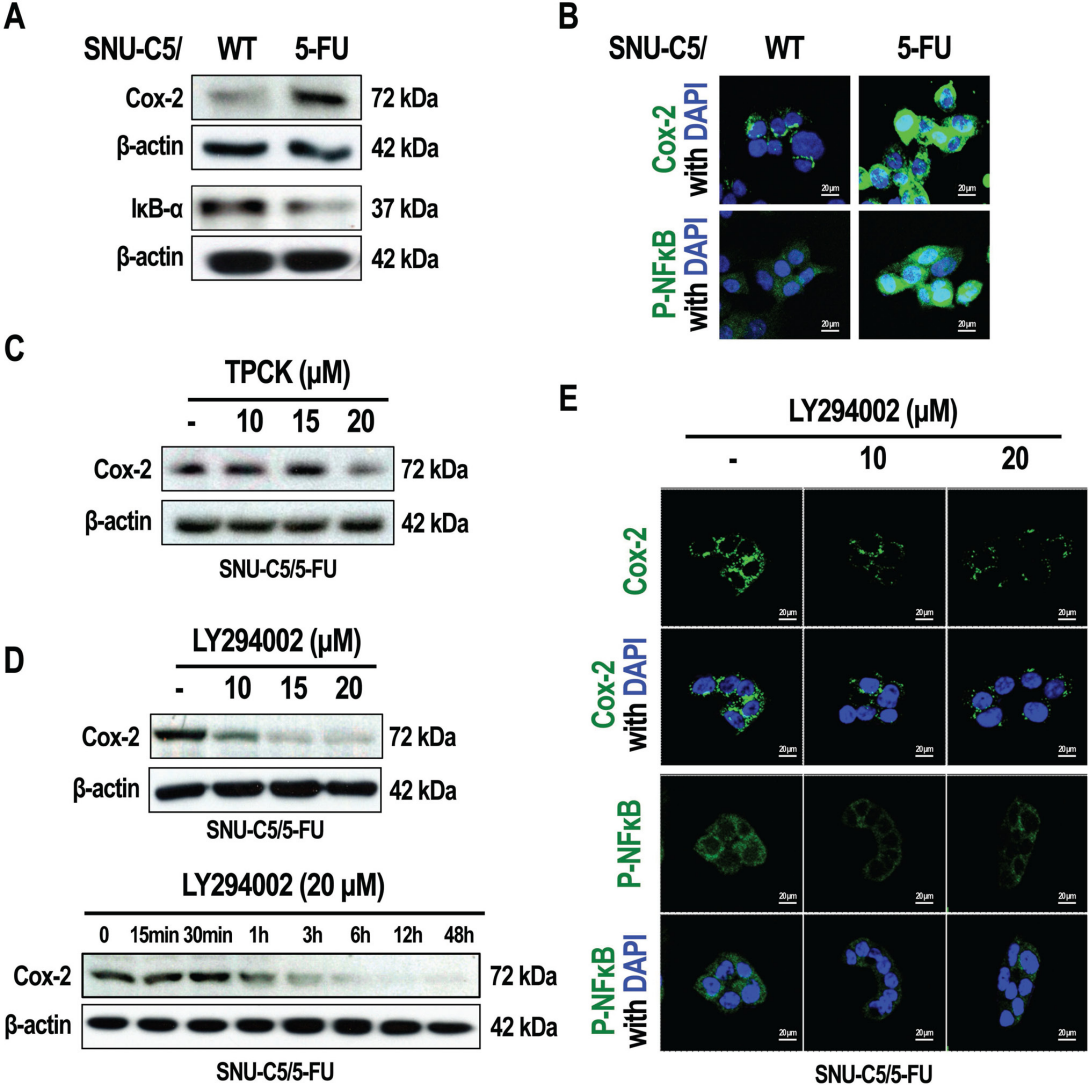
Inhibition of PI3K/AKT modulates NF-κB signaling in SNU-C5/5-FU cells (**A**) Immunoblot analysis of Cox-2 and IκB in SNU-C5/WT and SNU-C5/5-FU cells. (**B**) The localization of Cox-2 and P-NF-κB was confirmed by confocal microscopy. Scale bars, 20 μm. (**C**) Immunoblot analysis of Cox-2 in SNU-C5/5-FU cells treated with TPCK. (**D**) Immunoblot analysis of Cox-2 in SNU-C5/5-FU cells treated with LY294002. (**E**) The localization of Cox-2 and P-NF-κB was confirmed by confocal microscopy. Scale bars, 20 μm.

The NF-κB signaling pathway is involved in the promotion of cell survival. Phospho-AKT induces the activation of NF-κB signaling through the stimulation of IκB kinase [8–10]. On the other hand, NF-κB signaling also modulates COX-2 expression [13]. We therefore investigated activation of the NF-κB pathway in SNU-C5/5-FU and SNU-C5/WT cells by examining COX-2 expression levels. Therefore, compared to SNU-C5/WT cells, SNU-C5/5-FU cells were found to markedly overexpress COX-2 (Figure 4A and 4B). In addition, IκB-α levels were decreased, whereas phospho-NF-κB was overexpressed in SNU-C5/5-FU cells compared to SNU-C5/WT cells (Figure 4A and 4B). We also assessed whether the COX-2 overexpression occurred as result of signaling through the AKT pathway and the resultant activation of NF-κB signaling in SNU-C5/5-FU cells. When these cells were treated with TPCK (an IκB protease inhibitor), the COX-2 levels were found to decrease in a dose-dependent manner to levels similar to those seen in SNU-C5/WT cells (Figure 4C). We also found that the LY294002 decreased the levels of COX-2 in a dose- and time-dependent manner, as well as the levels of phospho-NF-κB (Figure 4D and 4E). These data show that activation of AKT induces the over-expression of COX-2 by activation of the NF-κB pathway in SNU-C5/5-FU cells.

### Activation of AKT leads to stabilization of cytoplasmic survivin via the overexpression of COX-2 in SNU-C5/5-FU cells

**Figure 5:**
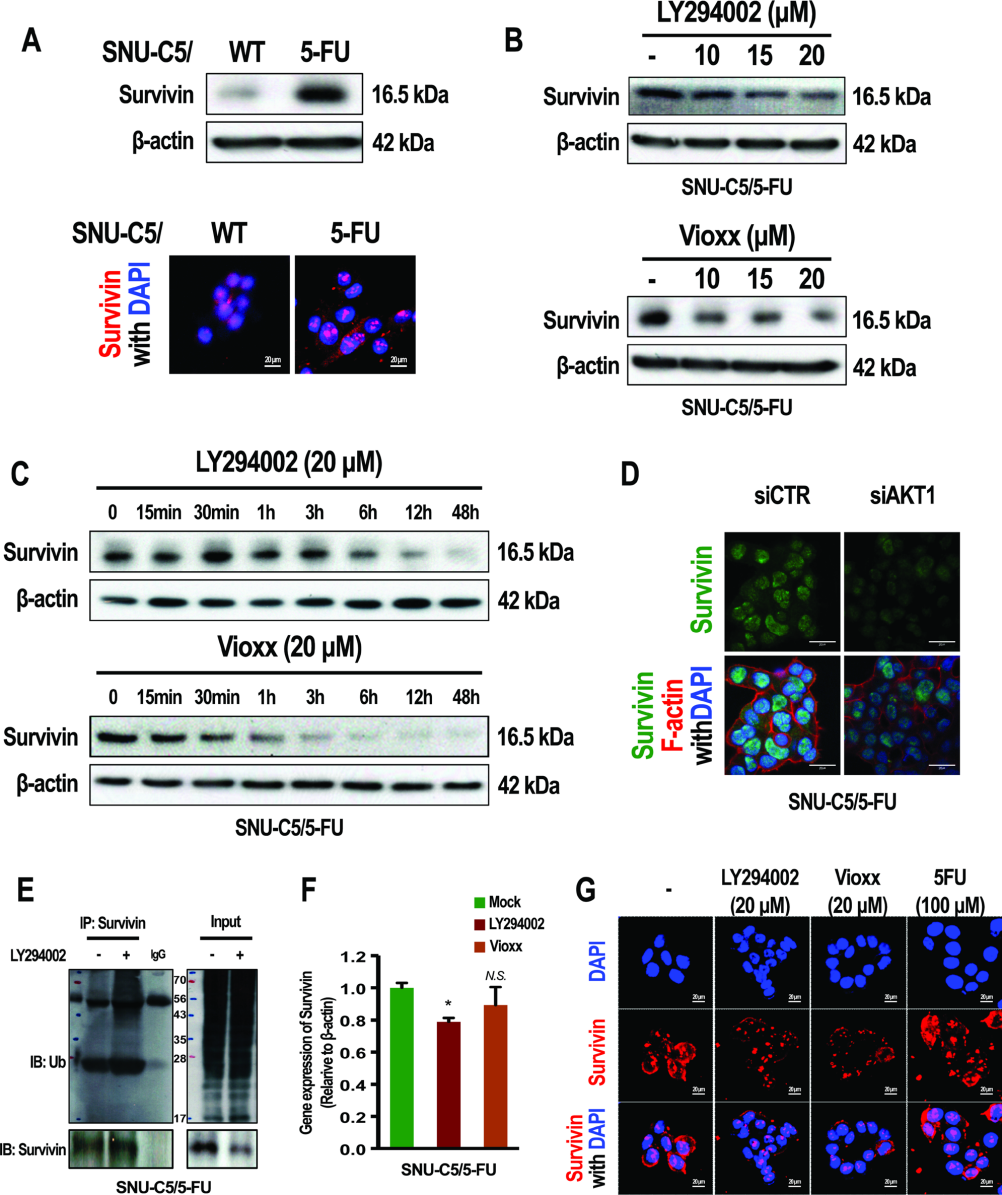
Effect of LY294002, Vioxxor or gene silencing of AKT1 on the expression of survivin in SNU-C5/5-FU cells (**A**, Top) Immunoblot analysis of survivin in SNU-C5/WT and SNU-C5/5-FU cells. (Bottom) The localization of survivin was confirmed by confocal microscopy. Scale bars, 20 μm. (**B**) Immunoblot analysis of survivin in SNU-C5/5-FU cells treated with different concentrations of LY294002 or Vioxx. (**C**) Immunoblot analysis of survivin in SNU-C5/5-FU cells treated with LY294002 or Vioxx for different periods of time. (**D**) For gene silencing of AKT1, SNU-C5/5-FU cells were transfected with the indicated amount of AKT1 siRNA or control siRNA. The localization of survivin and F-actin were confirmed by confocal microscopy. Scale bars, 20 μm. (**E**) Co-immunoprecipitation (IP) of survivin with ubiquitin in SNU-C5/5-FU cells treated with LY294002. (**F**) Real-time PCR (qPCR) measured survivin mRNA levels in SNU-C5/5-FU cells treatmented with the LY294002 (20 μM) or Vioxx (20 μM). (**G**) The localization of survivin was confirmed by confocal microscopy. Scale bars, 20 μm.

We next investigated how overexpression of COX-2, following the activation of AKT, induces anticancer drug resistance in SNU-C5/5-FU cells. The overexpression of PGE_2_ arising from the up-regulation of COX-2 has been previously reported in SNU-C5/5-FU cells [4]. In addition, PGE_2_ is known to induce resistance to apoptosis by inhibiting the ubiquitination of survivin in lung cancer cells [19]. We thus examined whether COX-2 levels could regulate the stabilization of survivin in SNU-C5/5-FU cells. Interestingly, survivin was found to be overexpressed in SNU-C5/5-FU cells compared with SNU-C5/WT cells (Figure 5A). LY294002 or Vioxx (a COX-2 selective inhibitor) decreased the levels of survivin in a dose- and time-dependent manner in SNU-C5/5-FU cells (Figure 5B and 5C). Also, gene silencing of AKT1 suppressed survivin expression (Figure 5D). This could be attributed to ubiquitination of survivin following treatment with LY294002 in SNU-C5/5-FU cells (Figure 5E). LY294002 and Vioxx greatly reduced mRNA level of survivin in SNU-C5/WT cells (Supplementary Figure 1D). In the SNU-C5/5-FU, the mRNA level of survivin was no affected or less affected by treatment of LY294002 and Vioxx (Figure 5F). These data indicate that activation of AKT induces the stabilization of survivin by upregulating COX-2 expression in SNU-C5/5-FU cells. Nuclear survivin has been reported to regulate the cell cycle, whereas cytoplasmic survivin induces resistance to apoptosis [19]. We observed that both nuclear and cytoplasmic survivin level were higher in SNU-C5/5-FU cells compared to SNU-C5/WT cells (Figure 5A). We noted that treatment with LY294002 or Vioxx decreased survivin levels in the cytoplasm rather than in the nucleus, whereas treatment with 5-FU did not affect survivin expression (Figure 5G). These data indicate that cytoplasmic levels of survivin are modulated by the activation of AKT through the overexpression of COX-2 in SNU-C5/5-FU cells.

### Inhibition of the PI3K/AKT pathway can overcome 5-FU resistance in SNU-C5/5-FU cells

**Figure 6:**
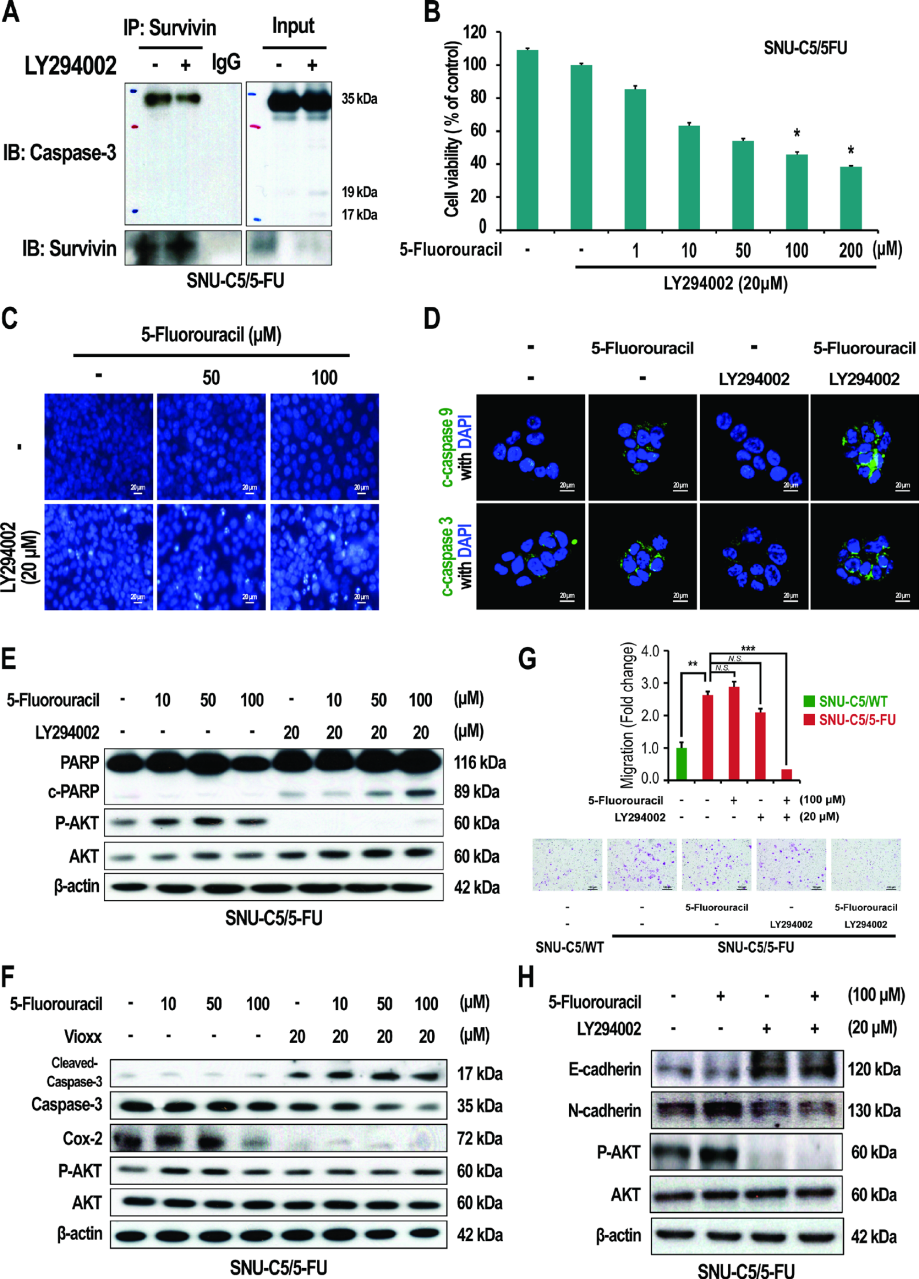
Effect of LY294002 and Vioxx on apoptosis and EMT in SNU-C5/5-FU cells (**A**) Co-immunoprecipitation (IP) of survivin with caspase-3 in SNU-C5/5-FU cells treated with LY294002. (**B**) The cytotoxicity of 5-FU with LY294002 on SNU-C5/5-FU cells was assessed using trypan blue staining. (**C**) SNU-C5/5-FU cells were stained with the DNA-specific fluorescent dye, Hoechst 33342. Apoptotic bodies were observed using an inverted fluorescent microscope equipped with an IX-71 Olympus camera. Scale bars, 20 μm. (**D**) The localization of cleaved-caspase-9 and cleaved-caspase-3 was confirmed by confocal microscopy. Scale bars, 20 μm. (**E**) Immunoblot analysis of PARP, cleaved-PARP, p-AKT, and AKT in SNU-C5/5-FU cells treated with 5-FU and/or LY294002. (**F**) Immunoblot analysis of cleaved-caspase-3, caspase-3, Cox-2, P-AKT, and AKT in SNU-C5/5-FU cells treated with 5-FU and/or Vioxx. (**G**) Effect of treatment of SNU-C5/5-FU with 5-FU and LY294002 on cell migration. Scale bars, 100 μm. The data are shown as the mean value ± SD from three independent experiments. ^*^p < 0.05 and ^**^p < 0.01 compared to the control. (**H**) Immunoblot analysis of E-cadherin, N-cadherin, P-AKT, and AKT in SNU-C5/5-FU cells treated with 5-FU and/or LY294002.

Cytoplasmic survivin contributes to resistance to apoptosis by interacting with caspase-3 or caspase-7 [30, 31]. We thus investigated if the increase in survivin, that arises as a result of AKT activation and increased COX-2 expression, could contribute to apoptosis resistance in SNU-C5/5-FU cells. First, we examined whether survivin interacts with caspase-3 in SNU-C5/5-FU cells. As shown in Figure 6A, in SNU-C5/5-FU cells, survivin was found to co-precipitate with caspase-3, and treatment with LY294002 reduced this interaction. These data suggest that activation of AKT allows SNU-C5/5-FU cells to avoid apoptosis through a direct interaction of caspase-3 with survivin.

We next addressed whether suppression of the PI3K/AKT pathway, or inhibition of COX-2, could attenuate the resistance to 5-FU seen in SNU-C5/5-FU cells. The viability of SNU-C5/5-FU cells decreased in a dose-dependent manner when co-treated with 5-FU and LY294002 (Figure 6B), compared to treatment with 5-FU only (Supplementary Figure 1E). In addition, co-treatment with 5-FU and Vioxx also decreased the viability of SNU-C5/5-FU cells (Supplementary Figure 1F). The IC_50_ values for co-treatment of the SNU-C5/5-FU cells were significantly reduced (the IC_50_ for co-treatment with LY294002 and 5-FU was 76.3 μM; the IC_50_ for co-treatment with Vioxx and 5-FU, was 97.4 μM; the IC_50_ for-FU treatment alone was 182.7 μM). When treated with 5-FU alone, apoptosis could not be observed. However, co-treatment with 5-FU and LY294002 in SNU-C5/5-FU cells increased chromatin condensation, a well-known characteristic of apoptotic cells (Figure 6C), along with the activation of apoptosis-related proteins such as increases in caspase-9 cleavage, caspase-3 cleavage, and poly(ADP-ribose)polymerase (PARP) cleavage (Figure 6D and 6E). In contrast, treatment with 5-FU alone did not activate caspase-3, whereas co-treatment with Vioxx and 5-FU increased caspase-3 cleavage (Figure 6F). These data indicate that inhibition of the PI3K/AKT pathway or COX-2 inhibition may overcome the resistance to 5-FU in SNU-C5/5-FU cells.

We also examined the epithelial-mesenchymal transition (EMT) in SNU-C5/5-FU cells. As a result, SNU-C5/5-FU cells showed enhanced migration compared with SNU-C5/WT cells. Co-treatment with LY294002 and 5-FU abrogated this enhanced migration of SNU-C5/5-FU cells (Figure 6G). Furthermore, treatment with LY294002, with or without 5-FU, attenuated the reduced levels of E-cadherin, an epithelial marker, and the enhanced levels of N-cadherin, a mesenchymal marker, in SNU-C5/5-FU cells (Figure 1B and 6H). These data indicate that over-activation of AKT pathway contributes to the EMT in SNU-C5/5-FU cells.

### Clinical significance and characterization of the gene expression profile in 5-FU resistance

**Figure 7:**
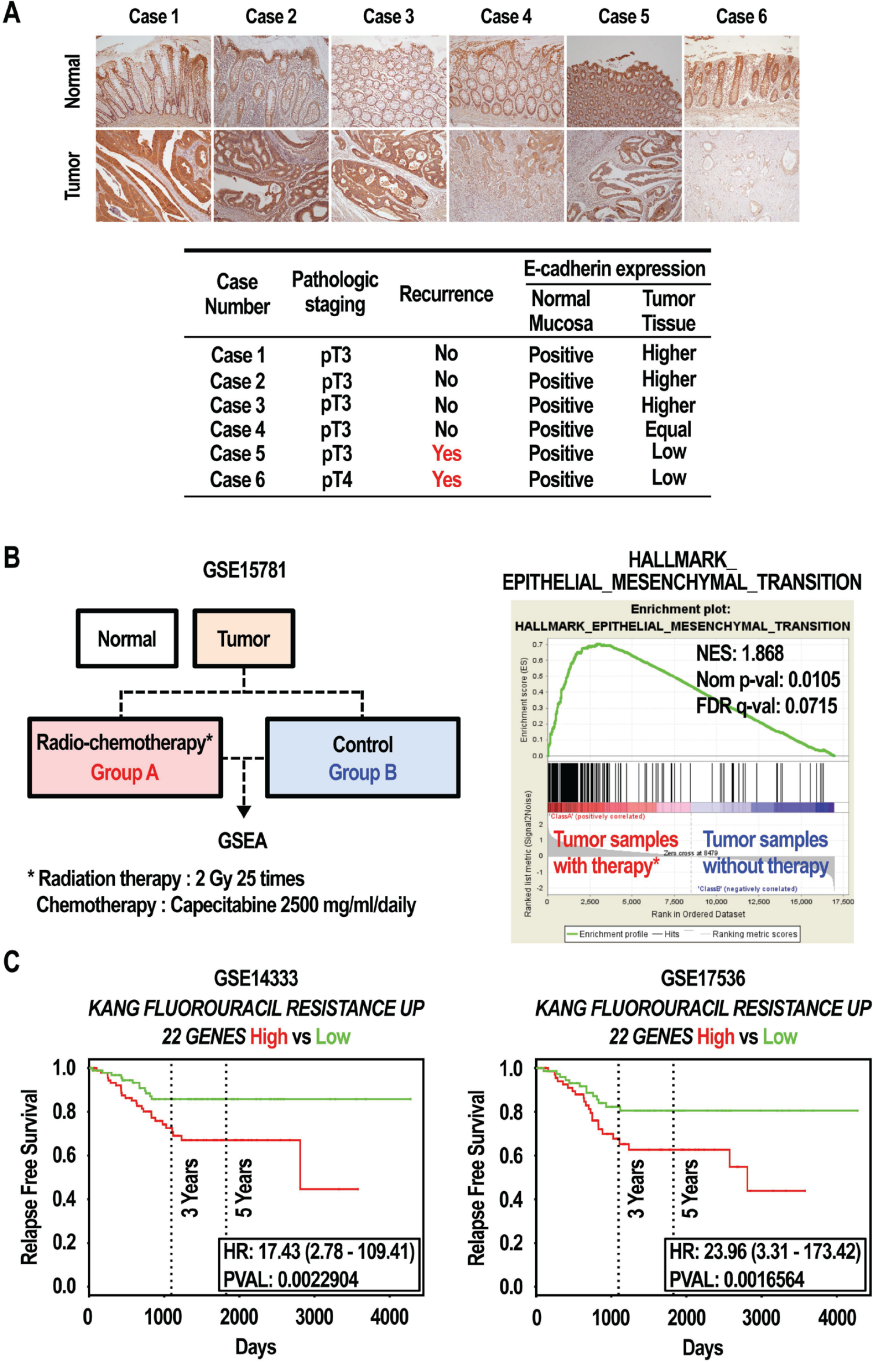
Effect of expression of E-cadherin in colon cancer patients (**A**) E-cadherin protein expression was assessed by IHC in tissues from colon cancer patients. E-cadherin protein in cancer tissues was lower in the patients with recurrence while equal or higher in the patients with no recurrence. (**B**) Gene expression by GSEA in colorectal cancer. GSEA analysis shows an enrichment of the EMT signature in colorectal cancer patients. The barcode indicates the position of genes related to EMT. NES: normalized enrichment score. (**C**) Survival rate by Kaplan–Meier (KM) plot in colorectal cancer. The relapse free survival curves were created using PROGgeneV2 with curated gene signature “KANG_FLUOROURACIL_RESISTANCE_UP (22 GENES)” in two different datasets with colorectal cancer, GSE14333 (*n* = 290) and GSE17536 (*n* = 177).

Compared with primary cancer patients, cancer patients with recurrence are likely to have developed anticancer drug resistance, and have an increased incidence of metastasis [32, 33]. The loss of E-cadherin expression promotes metastasis [34]. As shown in Figure 1A, E-cadherin was down-regulated in SNU-C5/5-FU cells compared to SNU-C5/WT cells. We thus investigated the expression of E-cadherin in tissues from colon cancer patients. Both normal and tumor tissues from colon cancer patients, with or without cancer recurrence, were stained with an E-cadherin specific antibody, and then the slides were interpreted by a pathologist. As shown in Figure 7A, E-cadherin expression was lower in tumor tissues from patients with cancer recurrence (case 6 and case 5) than in normal tissues. In contrast, E-cadherin levels were equal or higher in tumor tissues from patients without cancer recurrence (case 1, case 2, case 3, and case 4) than in normal tissues. Finally, we analyzed whether differences in the gene expression profile as a result of 5-FU treatment were important in the recurrence and survival of colon cancer patients. Using gene set enrichment analysis (GSEA), we found a strong positive correlation between genes upregulated in colorectal cancer patients treated with radio-chemotherapy (GSE15781) [35] and the EMT-related gene set (Figure 7B). Similar results in survival rate were obtained using the GSE14333 [36] and GSE17536 [37] expression data sets, independently. As shown in Figure 7C, patients with a high expression of the “KANG_FLUOROURACIL_RESISTANT_UP [38]” gene set had significantly poorer relapse-free survival (RFS) from both datasets (GSE14333, HR = 17.43, 95% CI 2.78–107.41, *P* = 0.0022904; GSE17536, HR = 23.96, 95% CI 3.31–173.42, *P* = 0.0016564). A survival analysis establishing subgroups by up-regulated genes via 5-FU resistance also revealed that genes whose expression profile was associated with 5-FU resistance may affect the survival rate in colon cancer patients (Figure 7C). These data show that EMT events, such as the loss of E-cadherin expression, might be an important mechanism in cancer recurrence and death in colorectal cancer patients with 5-FU resistance.

**Figure 8:**
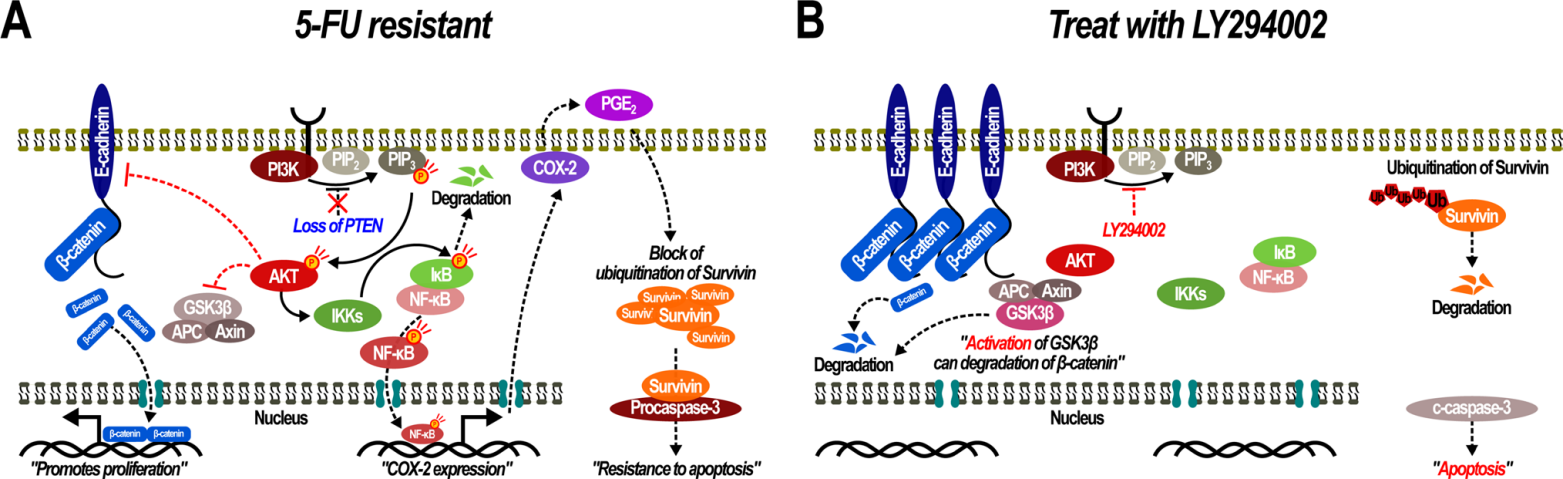
Acquiring and overcoming 5-fluorouracil resistance in SNU-C5/5-FU (**A**) Over-activation of PI3K/AKT leads to 5-FU resistance through activation of the β-catenin and NFκB pathways. (**B**) Inhibition of PI3K/AKT allows SNU-C5/5-FU cells to overcome their 5-FU resistance.

Collectively, in SNU-C5/5-FU cells, the increase in phospho-AKT levels, which arises from the loss of PTEN expression, modulates a variety of downstream effects such as nuclear translocation of β-catenin through loss of E-cadherin and inactivation of GSK-3β. Also, enhanced phospho-AKT levels lead to activation of NF-κB signaling, which results in stabilization of survivin in the cytoplasm and the inhibition of apoptosis (Figure 8A). Consequently, inhibition of AKT activation may overcome fluorouracil-resistance, and enhance 5-FU sensitivity sufficiently to cause apoptosis in SNU-C5/5-FU cells (Figure 8B).

## DISCUSSION

In this study, we investigated how cancer cells acquire or overcome resistance to anticancer drugs. To the best of our knowledge, this study is the first to demonstrate that over-activation of the AKT pathway is crucial for 5-FU resistance in SNU-C5/5-FU cells.

We have previously reported that the IC_50_ values following a 72-h treatment with 5-FU of SNU-C5/WT and SNU-C5/5-FU cells were 4.84 μM and 182.66 μM, respectively [39]. These data strongly indicated that SNU-C5/5-FU cells were resistant to 5-FU. 5-FU exerts its anti-cancer effects by inhibiting the biosynthesis of thymine. Several studies have shown that increased levels of TS lead to 5-FU resistance [3, 40, 41], whereas, interestingly, TS expression was not increased in SNU-C5/5-FU cells, suggesting a different mechanism for 5-FU resistance [4]. In this study, we found that compared to the parental SNU-C5/WT cells, SNU-C5/5-FU cells had important differences, which include higher levels of phospho-AKT, phospho-mTOR, nuclear β-catenin, COX-2, and survivin, and lower level of E-cadherin. In SNU-C5/5-FU cells, phospho-AKT regulates a variety of cellular processes including the down-regulation of E-cadherin, inactivation of GSK-3β, and activation of NF-κB signaling. Moreover, treatment with LY294002, a PI3 kinase inhibitor, led to not only reversal of these cellular processes but also enhanced apoptosis in SNU-C5/5-FU following 5-FU treatment.

The PI3K/AKT pathway is known to regulate cell growth and survival in cancer cells [5]. Moreover, phospho-AKT induces the inhibition of apoptosis via modulation of other cellular processes, including loss of E-cadherin through the activation of mdm2, an anti-apoptotic protein [7]. E-cadherin is a transmembrane protein, which is involved in cell-cell adhesion. The cytoplasmic domain of E-cadherin can bind to β-catenin, as well as to other proteins [25–27]. Thus, loss of E-cadherin leads to the release of β-catenin so that it can act as transcription factor to increase cell survival and proliferation [27]. In contrast, activation of GSK-3β induces the degradation of β-catenin in the cytoplasm. In this regard, it is well known that activation of AKT through its phosphorylation (phospho-AKT) leads to inactivation of GSK-3β [28, 42, 43]. In other words, phospho-AKT induces the down-regulation of E-cadherin and inactivation of GSK-3β, which is followed by an increase in the levels of the β-catenin transcription factor. We confirmed not only the down-regulation of E-cadherin in the plasma membrane, but also inactivation of GSK-3β in SNU-C5/5-FU cells (Figure 1A and 1B). We also noted that there were increased β-catenin levels in SNU-C5/5-FU cells compared to the SNU-C5/WT parental cells (Figure 1C and 1D). In the cytoplasm, increase in β-catenin levels leads to increased translocation into the nucleus, where it acts as a transcription factor to promote the expression of cell survival genes [27]. Interestingly, these series of events seem to be modulated by the increased phospho-AKT levels seen in SNU-C5/5-FU cells. Inhibition of AKT phosphorylation or gene silencing of AKT1 reduce release of plasma membrane bound β-catenin to the cytoplasm by stabilizing the E-cadherin/β-catenin complex at the plasma membrane and activating GSK-3β (Figure 2). Thus, inhibition of the PI3K/AKT pathway is likely to suppress β-catenin action as a transcription factor in SNU-C5/5-FU cells.

Previous studies have reported that loss of PTEN could explain the increased phosphorylation of AKT observed in colorectal cancer cells [29, 44]. In this study, we found that the expression of PTEN was down-regulated in SNU-C5/5-FU cells compared with SNU-C5/WT cells (Figure 3A and 3B). SNU-C5/WT cells treated with a PTEN targeted siRNA (si-PTEN) had the same phenotype as SNU-C5/5-FU cells, such as reduced E-cadherin and β-catenin levels at the plasma membrane (Figure 3E and 3F), and increased β-catenin expression in the cytoplasm and nucleus (Figure 3F). These results indicate the possibility that the 5-FU resistance that arises as a result of increased phospho-AKT levels is because of the loss of PTEN in SNU-C5/5-FU cells.

The EMT is defined as the conversion of epithelial cells into a mesenchymal phenotype. In cancer cells, the EMT is associated with increased invasive and metastatic potential [45]. We found that SNU-C5/5-FU cells had a down-regulation of E-cadherin, an epithelial marker (Figure 1A and 1B), compared to SNU-C5/WT cells. Loss of E-cadherin is known to promote the EMT and metastasis [34]. In fact, our previous studies on 5-FU resistance focused exclusively on the interaction between E-cadherin and β-catenin, and the association of E-cadherin and AKT. On the other hand, the EMT also plays a critical role in anticancer drug resistance [46]. The decreased E-cadherin that occurs because of AKT over-activation shows the association between EMT and 5-FU resistance in SNU-C5/5-FU cells. Previous studies have shown that recurrent cancer is more resistant to anticancer drugs and has greater metastatic ability than primary cancer [32, 33]. Similarly, we found that the E-cadherin level was down-regulated in recurrent colon cancer tissues compared to primary colon cancer tissues (Figure 7A). Treatment with LY294002 abrogated the down-regulation of E-cadherin, an epithelial marker, and the enhanced level of N-cadherin, a mesenchymal marker, in SNU-C5/5-FU cells (Figure 6H). The 5-FU resistance of SNU-C5/5-FU cells was involved in the EMT, leading to increased migratory capacity in SNU-C5/5-FU cells. Treatment with LY294002 suppressed this increased migratory capacity of SNU-C5/5-FU cells (Figure 6G). Furthermore, we confirmed that the gene expression pattern in patients treated with radio-chemotherapy is positively correlated with the genes related to the EMT. Similar to our GSEA using GSE15781, there are studies that show that radiation or chemo-resistance to oxaliplatin can induce the EMT in CaR1 or DLD1 colon cancer cells [47, 48]. Although it is not from 5-FU single treatment, our data shows that 5-FU resistance is an important mechanism in the induction of EMT in colon cancer. In particular, consistent with our results, *CDH1* is one of the top 50 genes negatively regulated in patients undergoing radio-chemotherapy (data not shown). This data suggests that E-cadherin (*CDH1*) expression may be an important factor for the induction of EMT by 5-FU resistance. Furthermore, we investigated whether the survival rate of colon cancer patients is related to the gene signature associated with 5-FU resistance. Although the gene signature used in this analysis is from gastric cancer cells, and the number of patients is the two datasets is small, we noted that the rate of RFS was lower in patients with a high level of expression of genes associated with 5-FU resistance in the two datasets (GSE14333 and GSE17536). Overall, these bioinformatic data strongly support our result that a change in E-cadherin (*CDH1*) expression may be an important mechanism in cancer recurrence and/or the death of colon cancer patients as a result of 5-FU resistance. It is therefore reasonable to propose that in SNU-C5/5-FU cells, the EMT that occurs as a result of the loss of E-cadherin expression is another mechanism causing 5-FU resistance.

Various studies have shown that phospho-AKT promotes the activation of NF-κB signaling by activating IκB kinase, which then results in the induction of COX-2 expression [8–10]. SNU-C5/5-FU cells had increased levels of COX-2 and increased NF-κB signaling compared with SNU-C5/WT parent cells (Figure 4A and 4B). Furthermore, inhibition of PI3K/AKT, reduced not only NF-κB activation, but also COX-2 expression (Figure 4D and 4E). It has been reported that COX-2 modulates resistance to apoptosis by inhibiting the ubiquitination of survivin, an anti-apoptotic protein [19]. We thus inferred that activation of NF-κB by phospho-AKT could mediate resistance to apoptosis through survivin. Survivin was found to be overexpressed in SNU-C5/5-FU cells compared with SNU-C5/WT parental cells, and of inhibition of PI3K/AKT or COX-2 decreased survivin levels in a time- and dose- dependent manner by promoting its ubiquitination (Figure 5). Survivin is known to suppress apoptosis and promote the development of tumor cells by modulating microtubule dynamics [20]. Survivin modulates the cell cycle in the nucleus, whereas in the cytoplasm, survivin directly suppresses both caspase-3 and caspase-7, leading to resistance to apoptosis [30, 31]. It is known that PGE_2,_ which is synthesized as a result of COX-2 activity, stabilizes survivin in the cytoplasm [19]. Our data demonstrated that in SNU-C5/5-FU cells, increased phospho-AKT levels leads to the overexpression of COX-2, thereby increasing the survivin levels in the cytoplasm. As mentioned above, several studies showed that stabilized survivin contributes to resistance to apoptosis by interacting with caspase-3 or caspase-7 [30, 31]. We found that inhibition of either PI3K/AKT or COX-2 decreased the interaction with survivin and procaspase-3, suggesting a correlation between phospho-AKT levels and survivin in SNU-C5/5-FU cells. As mentioned above, phospho-AKT indirectly induces COX-2 expression through activation of NF-κB signaling leading to increased expression of COX-2. This increased COX-2 expression results in an increase in PGE_2_ levels and contributes to the stabilization of survivin. In the cytoplasm, the increased levels of survivin induce resistance to apoptosis by interacting with procaspase-3 (Figure 8A). As a result, inhibition of PI3K/AKT removed this resistance to apoptosis by promoting the degradation of survivin (Figure 6).

On the other hand, to address whether only 5-FU-resistant colorectal cancer cells show over-activation of AKT signaling, we compared SNU-C5/5-FU and SNU-C5/OXT, an oxaliplatin-resistant cell line. Remarkably, SNU-C5/5-FU showed the over-activation of AKT and the increased expression of COX-2 and survivin, but not SNU-C5/OXT (data not shown). Interestingly, it has been reported that various types of cancer have several pathways for 5-FU resistance. In the case of 5-FU-resistant breast cancer cells, 5-FU induced the expression of ADAM12-L, isoform of a disintegrin and metalloproteases (ADAMs). In addition, overexpression of ADAM12-L resulted in over-activation of PI3K/AKT signaling and reduced sensitivity to 5-FU [49]. The overexpression of RhoGDI2, one of a family of Rho GTPase dissociate inhibitors (GDIs), contributed to 5-FU resistance in gastric cancer cells [50]. BAF57 which mediates direct interactions with estrogen and androgen receptors, reduced sensitivity to anticancer agent including 5-FU in ovarian cancer cells [51]. Depending on the types of cancer cells, 5-FU-resistant cancer cells seem to acquire resistance to 5-FU through various pathways. In present study, we demonstrated that over-activation of AKT led to characteristics of 5-FU resistance such as induction of EMT and the stabilization of survivin and β-catenin in SNU-C5/5-FU cells.

Taken together, compared to SNU-C5/WT parental cells, SNU-C5/5-FU cells over-express phospho-AKT as a result of loss of PTEN. The over-activated AKT pathway that results from loss of PTEN contributes to 5-FU resistance by regulating the expression of a variety of downstream molecules such as E-cadherin and GSK-3β, and increasing NF-κB signaling in SNU-C5/5-FU cells (Figure 8A). Inhibition of PI3K/AKT allows SNU-C5/5-FU cells to overcome their 5-FU resistance leading to the induction of apoptosis by 5-FU (Figure 8B). These data demonstrate that inhibition of PI3K/AKT could be a good therapeutic target to treat anti-cancer drug resistant colon cancer.

## MATERIALS AND METHODS

### Reagents

Hoechst 33342, 5-FU, and trypan blue were purchased from Sigma (Sigma Chemical Co., St. Louis, MO, USA). Mouse monoclonal anti-E-cadherin, anti-PTEN, anti-α tubulin, and anti-ubiquitin antibodies, rabbit polyclonal anti-caspase-3, anti-β-catenin, and anti-IκB-α antibodies, and goat polyclonal anti-COX-2 and anti-survivin antibodies were purchased from Santa Cruz Biotechnology (Santa Cruz Biotech, CA, USA); rabbit monoclonal anti-p-NF-κB, anti-GSK-3β, and anti-cleaved caspase-3 antibodies, rabbit polyclonal anti-phospho-GSK-3β, anti-AKT, anti-phospho-AKT, anti-cleaved caspase-9, anti-phospho-mTOR, anti-mTOR, anti-N-cadherin, and anti-PARP antibodies were purchased from Cell Signaling Technology (Cell Signaling Technology, Beverly, MA, USA); the mouse monoclonal anti-cyclin D1 antibody was purchased form BD Biosciences (BD Biosciences, San Jose, CA, USA); the mouse monoclonal anti-β-actin antibody was purchased from Sigma (Sigma Chemical Co., St. Louis, MO, USA). Secondary HRP conjugated anti-mouse, anti-goat, and anti-rabbit antibodies were purchased from Santa Cruz Biotechnology (Santa Cruz Biotech, CA, USA). LY294002 was purchased from Calbiochem (Merck KGaA, Darmstadt, Germany). Vioxx was purchased from Santa Cruz Biotechnology (Santa Cruz Biotech, CA, USA). Dynabeads^®^ Protein G was purchased from NOVEX^®^ (Invitrogen, Carlsbad, CA). Aprotinin, leupeptin, Nonidet P-40 were obtained from Roche (Roche Applied Science, Indianapolis, IN, USA). The western blotting reagent West-Zol was obtained from Intron (iNtROn Biotechnology, Gyeonggi-do, Korea). VECTASHIELD antifade mounting medium containing DAPI was purchased from Vector Laboratories (Burlingame, CA, USA).

### Cell culture

SNU-C5/WT, a human colon cancer cell line, was obtained from the Korean Cell Line Bank (KCLB). SNU-C5/5-FU cells, a 5-fluorouracil-resistant human colon cancer cell line, was obtained from the Research Center for Resistant Cells. SNU-C5/WT and SNU-C5/5-FU cells were cultured in RPMI 1640 (Hyclone, Logan, UT, USA) medium supplemented with 10% heat inactivated fetal bovine serum (Hyclone), 100 U/mL penicillin, and 100 mg/mL streptomycin (GIBCO Inc., Grand Island, NY, USA) at 37°C in a humidified atmosphere with 5% CO_2_. For SNU-C5/5-FU cells, the medium containing 140 μM 5-FU was changed after 48 h.

### Cell viability assay

The effect of 5-fluorouracil on the growth of SNU-C5/WT and SNU-C5/5-FU cells was evaluated using trypan blue staining. Co-treatment of 5-FU with LY294002 (a PI3 kinase inhibitor) or with Vioxx (a COX-2 selective inhibitor) on the growth of SNU-C5/5-FU cells was evaluated using trypan blue staining [52]. Cells were seeded at 2 × 10^5^ cells/mL in 24-well plates at 37°C in 5% CO_2_ gas to allow cell attachment. After 24 h, the cells were treated with 5-FU (1, 10, 50, 100, and 200 μM) or/and LY294002 (20 μM) or Vioxx (20 μM) for 72 h. At the end of the experimental incubation, cells were detached using 0.25% trypsin-EDTA. Cell pellets were then suspended in PBS and 100 μL of the resuspended cells were mixed with an equal volume of 0.01% trypan blue solutions for 4 min. Unstained cells (viable cells) in the mixture were counted using a hemocytometer. Each experiment was repeated at least three times. Concentration (X-axis)-response (% control optical density; Y-axis) curves were obtained. We determined the IC_50_ value (compound concentration resulting in 50% inhibition of growth).

### Morphological analysis of apoptosis by Hoechst 33342 staining

SNU-C5/5-FU cells were seeded at 2 × 10^5^ cells/mL in 1 mL in 24-well microplates. After 24 h of incubation, cells were treated with LY294002 (20 μM) and/or 5-FU (100 μM) for 24 h. The cells were incubated with Hoechst 33342 (10 μg/mL final concentration in medium) at 37° C for 30 min. SNU-C5/5-FU cells were observed using an inverted fluorescent microscope equipped with an IX-71 Olympus camera and photographed (magnification ×20).

### Western blot analysis

SNU-C5/WT and SNU-C5/5-FU cells were seeded at 2 × 10^5^ cells/mL. After 24h, the cells were lysed with lysis buffer (50 mM Tris-HCl [pH 7.5], 150 mM NaCl, 2 mM EDTA, 1 mM EGTA, 1 mM NaVO_3_, 10 mM NaF, 1 mM dithiothreitol, 1 mM phenylmethylsulfonylfluoride, 25 μg/mL aprotinin, 25 μg/mL leupeptin, 1% Nonidet P-40) for 30 min at 4° C. SNU-C5/5-FU cells were then seeded at 2 × 10^5^ cells/mL and culture for 24 h and then treated with LY294002 or Vioxx (20 μM) and/or 5-FU (10, 50 and 100 μM) for 15 min–24 h. After treatment, SNU-C5/5-FU cells were lysed with lysis buffer for 30 min at 4° C. The lysates were centrifuged at 15,000 rpm, at 4° C for 15 min. Protein content was determined according to the method of Bradford assay [53]. The cell lysates were separated using 6∼15% SDS-PAGE gels and then separated proteins were then transferred to polyvinylidene fluoride membrane (Bio-Rad, Hercules, CA, USA) in glycine transfer buffer (192 mM glycine, 25 mM Tris-HCl [pH 8.8], and 20% MeOH [v/v]) at 200 mA for 2 h. After blocking with 5% skimmed milk solution, the membrane was incubated with primary antibodies against PARP (1:2000), caspase-3 (1:1000), cleaved caspase-3 (1:1000), caspase-9 (1:1000), cleaved caspase-9 (1:1000), AKT (1:1000), phospho-AKT (1:1000), GSK-3β (1:1000), phospho-GSK-3β (1:1000), β-catenin (1:2000), E-cadherin (1:1000), mTOR (1:1000), phospho-mTOR (1:1000), phospho-NF-κB (1:1000), IκB (1:1000), COX-2 (1:1000), survivin (1:1000), PTEN (1:1000), ubiquitin (1:1000), cyclin D1 (1:1000), α-Tubulin (1:1000) and β-actin (1:5000) at 4° C overnight, and incubated with the appropriate HRP conjugated secondary antibody (1:5000) at room temperature for 1 h. Protein bands were detected using a WEST-ZOL^®^ plus Western Blot Detection System (iNtRON Biotechnology iNtRON., Gyeonggi-do, Korea) with subsequent exposure to X-ray films (AGFA, Mortsel, Belgium).

### Co-immunoprecipitation assay

SNU-C5/5-FU cells were seeded at 2 × 10^5^ cells/mL for 24 h and treated with LY294002 (20 μM) for 24 h. After treatment, SNU-C5/5-FU cells were lysed with lysis buffer for 30 min at 4° C. The lysates were centrifuged at 15,000 rpm, at 4° C for 15 min. Dynabeads^®^ Protein G (50 μL) were added to the tube and removed from the supernatant by placing the tube on a magnet to separate the beads. Separated beads were the added directly to antibody in 200 μL of PBS containing 0.02% Tween-20 and incubated with rotation for 10 min at room temperature. The supernatant was then removed. The bead-antibody complex was washed using 200 μL of PBS containing 0.02% Tween-20 and the supernatant removed. The bead-antibody complex was added directly to the cell lysates and incubated by rotation for 10 min at room temperature. The supernatant was removed and the bead-antibody-Ag complex was washed three times using 200 μL of PBS containing 0.02% Tween-20 and the supernatant removed. Elution buffer (20 μL, 50 mM Glycine [pH 2.8]) was added to the bead–antibody–Ag complex along with 10 μL of NuPAGE LDS sample buffer and then heated for 100 min at 70° C. The supernatant was separated from the beads using a magnet and loaded onto an SDS-PAGE gel.

### Migration assay

The migration assay was performed using a trans-well (Neuro Probe, Inc., Gaithersburg, MD, USA) coated with fibronectin (10 μg/mL). Cells were suspended in serum-free medium, and added to the upper chamber of the trans-well inserts. Medium with 3% fetal bovine serum was then added to the lower chamber. After incubation for 12 h, non-migrated cells on the upper surface of the membrane were scrapped off, and the migrated cells on the lower surface were stained using Diff-quick, and then counted using four randomly chosen high power fields (20 × magnification). All experiments were repeated at least three times with two replicates each.

### Confocal microscopy

Cells were fixed in 3.5% formaldehyde for 30 min at room temperature (RT). Following this, the cells were permeabilized with 0.1% Triton X-100 for 10 min at RT. The cells were then blocked in 3% BSA for 1 h at RT. The cells were then treated with primary antibodies (1:100) overnight at 4° C. Immunofluorescent staining of primary antibodies was achieved by staining with Alexa Fluor 488 goat anti-rabbit IgG, Alexa Fluor 488 goat anti-mouse IgG, or Alexa Fluor 594 rabbit anti-goat IgG secondary antibodies, as appropriate (BioLegend, San Diego, CA, USA). The stained images were visualized using an FV500 confocal microscope (Olympus, Tokyo, Japan).

### Gene silencing

PTEN siRNA (sc-29459) and AKT1 siRNA (sc-29195) were purchased from Santa Cruz Biotechnology. SNU-C5/WT cells were transfected with siRNA using Lipofectamine^™^ 2000 Transfection reagent (Invitrogen) following the manufacturer's protocol. The ratio of siRNA versus Lipofectamine reagent was 1:1.15. After 4 h of transfection, the cells were used in further experiments.

### RNA preparation and real-time quantitative polymerase chain reaction (RT-qPCR)

Total RNA was prepared using TRIzol^®^ RNA isolation reagents (Invitrogen) according to the manufacturer's instructions. Reverse transcription was performed with a First Strand cDNA Synthesis kit (Promega, Madison, WI). This cDNA used as a template for real-time quantitative PCR (RT-qPCR). Also, RT-qPCR was performed with an iQ^™^ SYBR^®^ Green Supermix (Bio-Rad, Hercules, CA) with a CFX384 Real-Time PCR (Bio-Rad). RT-qPCR results were expressed using the CFX Manager software (Bio-Rad) that measures amplification of the target and the endogenous control in experimental samples and in a reference sample. Measurements were normalized using the endogenous control. The primer sequences were used in RT-qPCR are shown in below: primer sequences 5′-TGA CGA CCC CAT AGA GGA ACA -3′ (forward) and 5′-CGC ACT TTC TCC GCA GTT TC-3′ (reverse) for BIRC5 (Survivin), 5′-TGA AGC TGA GGG AGC CAC AGC -3′ (forward) and 5′-GGG TTC TCC CTG GGC ACC AA-3′ (reverse) for β-catenin, 5′-TGC CCA GAA AAT GAA AAA GG-3′ (forward) and 5′-GTG TAT GTG GCA ATG CGT TC (reverse) for E-cadherin, 5′-GTG GGG CGC CCC AGG CAC CA-3′ (forward) and 5′-CTC CTT AAT GTC ACG CAC GAT TTC (reverse) for β-actin.

### Immunohistochemical (IHC)-staining

The bio-specimens and data used for this study were provided by the Biobank of Jeju National University Hospital, a member of the Korea Biobank Network (A-03-03). Immunohistochemistry was performed on 4-μm-thick tissue sections. Antigen retrieval for E-cadherin was achieved by heat treatment at 95° C for 20 min. Before staining the sections, endogenous peroxidase was blocked. The primary antibody was E-cadherin (1:50; Santa Cruz Biotechnology, Inc.) and the incubation was carried out overnight at 4° C. The reaction was visualized using the Streptavidin-Biotin Complex (Dako, Glostrup, Denmark). Sections were counterstained with hematoxylin.

### Statistical analysis

Results are shown as means ± standard deviation (SD) from three independent experiments. Student's *t*-test was used to analyze the data with the following significance levels: ^*^p < 0.05, ^**^p < 0.01, ^***^p < 0.001. All assays were performed with at least three independent experiments.

## SUPPLEMENTARY MATERIALS FIGURE


