# Supplementary material for: Over-activation of AKT signaling leading to 5-Fluorouracil resistance in SNU-C5/5-FU cells
Source: Oncotarget. 2018 Apr 13;9(28):19911–28. doi: 10.18632/oncotarget.24952 (PMC5929436; doi:10.18632/oncotarget.24952)
Supplement: Supplementary file 1 [file oncotarget-09-19911-s001.pdf]

Over-activation of AKT signaling leading to 5-Fluorouracil resistance in SNU-C5/5-FU cells

SUPPLEMENTARY MATERIALS

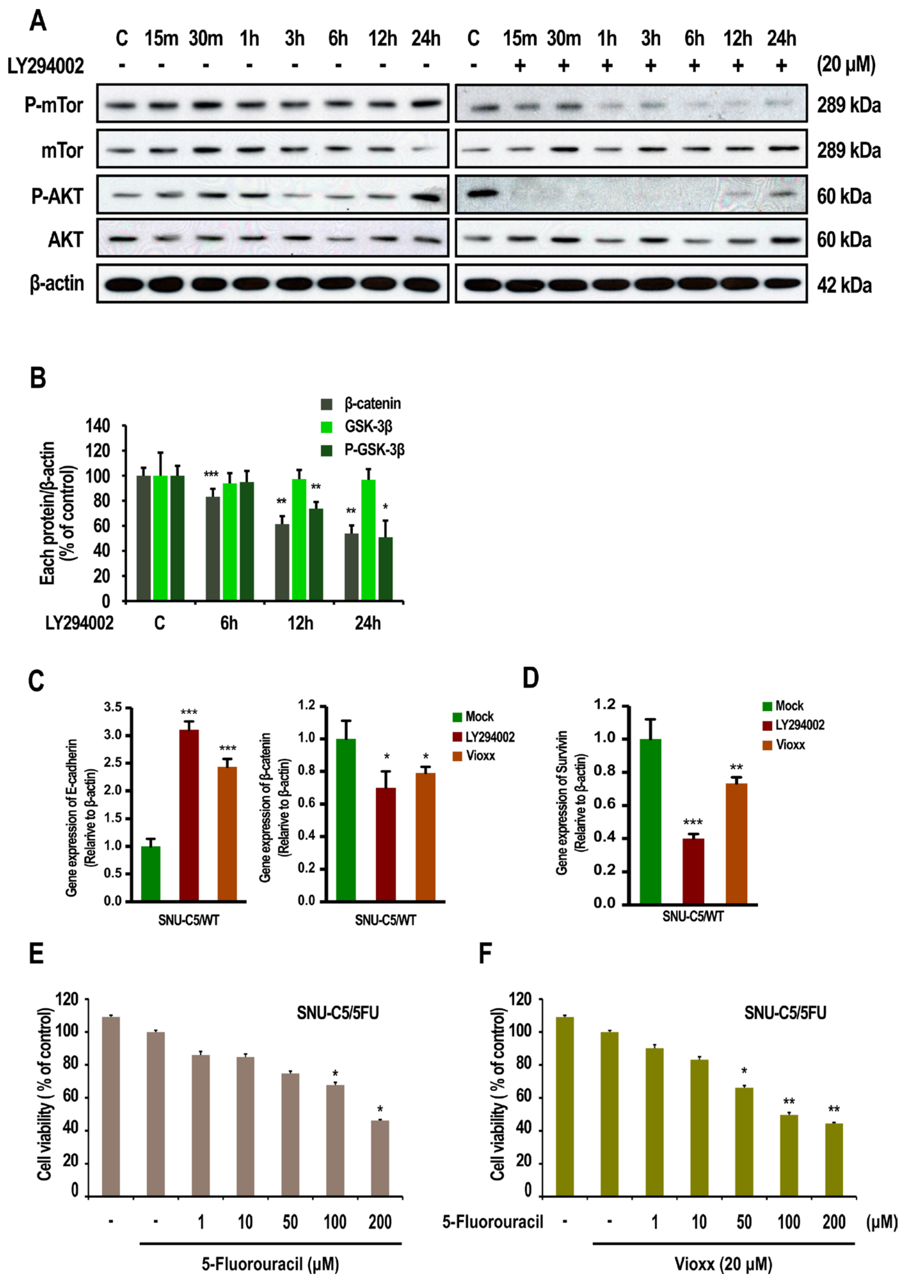

Supplementary Figure 1: (**A**) Immunoblot analysis of P-mTor, mTor, P-AKT, and AKT in SNU-C5/5-FU cells treated with LY294002 (20 μM). (**B**) Data represent the percentage of E-cadherin expression in SNU-C5/5-FU cells. The data are presented as the mean value ± SD from three independent experiments. $^{*}p < 0.05$ and $^{**}p < 0.01$ compared with the control. (**C**) Real-time PCR (qPCR) measured E-cadherin and β-catenin mRNA levels in SNU-C5/WT cells treatmented with the LY294002 (20 μM) or Vioxx (20 μM). (**D**) Real-time PCR (qPCR) measured survivin mRNA levels in SNU-C5/WT cells treatmented with the LY294002 (20 μM) or Vioxx (20 μM). (**E**) The cytotoxicity of 5-FU on SNU-C5/5-FU cells was assessed using trypan blue staining. (**F**) The cytotoxicity of 5-FU with Vioxx on SNU-C5/5-FU cells was assessed using trypan blue staining.
